# The Role of Microglia in Diabetic Retinopathy: Inflammation, Microvasculature Defects and Neurodegeneration

**DOI:** 10.3390/ijms19010110

**Published:** 2018-01-01

**Authors:** Christine Altmann, Mirko H. H. Schmidt

**Affiliations:** Molecular Signal Transduction Laboratories, Institute for Microscopic Anatomy and Neurobiology, Johannes Gutenberg University, School of Medicine, 55131 Mainz, Germany; Christine.Altmann@unimedizin-mainz.de

**Keywords:** microglia, retina, diabetic retinopathy, neurodegeneration, angiogenesis

## Abstract

Diabetic retinopathy is a common complication of diabetes mellitus, which appears in one third of all diabetic patients and is a prominent cause of vision loss. First discovered as a microvascular disease, intensive research in the field identified inflammation and neurodegeneration to be part of diabetic retinopathy. Microglia, the resident monocytes of the retina, are activated due to a complex interplay between the different cell types of the retina and diverse pathological pathways. The trigger for developing diabetic retinopathy is diabetes-induced hyperglycemia, accompanied by leukostasis and vascular leakages. Transcriptional changes in activated microglia, mediated via the nuclear factor kappa-light-chain-enhancer of activated B cells (NFκB) and extracellular signal–regulated kinase (ERK) signaling pathways, results in release of various pro-inflammatory mediators, including cytokines, chemokines, caspases and glutamate. Activated microglia additionally increased proliferation and migration. Among other consequences, these changes in microglia severely affected retinal neurons, causing increased apoptosis and subsequent thinning of the nerve fiber layer, resulting in visual loss. New potential therapeutics need to interfere with these diabetic complications even before changes in the retina are diagnosed, to prevent neuronal apoptosis and blindness in patients.

## 1. Introduction

Diabetic retinopathy (DR) is one of the most common complications of diabetes [1] and the main cause of vision impairment and loss in individuals from 20 to 74 years of age [2,3]. After suffering 20 years with diabetes, nearly all patients with type 1 and more than 60% of patients with type 2 diabetes develop a retinopathy [4,5,6] and the number of patients extensively increases every year [7]. The main cause of the complication remains unclear. Symptoms of DR range from hemorrhages, micro-aneurysms, cotton-wool spots, lipid exudates, macular edema, capillary occlusion and neovascularization and ultimately blindness [8].

Clinically, DR can be divided into two forms: non-proliferating diabetic retinopathy (NPDR) and proliferating diabetic retinopathy (PDR) [9]. NPDR is the first stage of DR and is characterized by damage to retinal vasculature, increased vascular permeability, thickening of the basement membrane, loss of pericytes and the beginning of mobilization of blood vessels. It can be divided into mild (micro-aneurysms), moderate (micro-aneurysms, retinal hemorrhages or hard exudates), severe (20 hemorrhages in each of the four quadrants, venous beading in two quadrants or intra-retinal microvascular anomalies) and very severe (combined complications) phases [9,10,11]. NPDR can further progress into PDR, which is defined by pathological neovascular growth (angiogenesis), vitreous hemorrhage, retinal scars and detachment, resulting in irreversible vision loss and total blindness [11,12]. Diabetic macular edema (DME), a side effect of both NPDR and PDR, can occur at any stage of DR and is caused by increased vascular permeability and leakage of proteins and lipids into the extracellular space. The functional damage of the retinal vascular epithelium results in an increase of extracellular fluid, which, collected by Müller cells, leads to swelling of the retina and increased cell death, further resulting in visual impairment and loss [13,14,15].

DR was first described as a classically microvascular disease, based on the discovery of the microvascular changes in the retina and for an extended period, DR was solely a microvascular abnormality. It was gradually discovered that all major cell types of the retina are altered and today DR is believed to result from interplay between endothelial cells, microglia, astrocytes, Müller cells and neurons. Thus, DR is a microvascular disease as well as a chronic inflammation and retinal neurodegeneration, which is not only influenced by local changes, but also by systemic metabolic and cardiovascular parameters. Degeneration, inflammation and vascular alternations occur and operate parallel and in close relation.

In this review, the inflammatory component of the disease is examined carefully and summarized regarding the role of microglia cells in neurodegeneration of retinal neurons. Inflammation means tissue invasion of immune cells to clear after an infection or damage of tissue. The retina was long believed to be an immune-privileged tissue. However, it was discovered that several cytokines and chemokines are increased in the diabetic retina [16], which resulted in growing evidence that inflammation and microglia play a major role in the development and progression of DR, as well as in retinal neurodegeneration [17,18,19]. Microglia are the resident inflammatory cells of the central nervous system (CNS), which upon activation can modulate inflammatory processes and are involved in a variety of other neuroinflammatory and neurodegenerative diseases, such as Alzheimer’s, Parkinson’s and Huntington’s diseases [20,21].

Recently, functional alterations in patients with DR were described, which occurred even before development of vascular dysfunction, indicating that diabetes-induced hyperglycemia has a vasculature-independent effect on the neuronal retina [22,23]. In patients with diabetes, but without retinopathy, defects in the electroretinogram (ERG) were detected as were neurodegeneration and a thinning of the innermost layer, the nerve fiber layer, suggesting a loss of axons [19,24,25]. The link between hyperglycemia and neurodegeneration is not fully understood, however, various cues point to altered inflammation as a link and activation of retinal microglia as an early feature of DR [26].

Despite the progress made in the last years, understanding the pathogenesis of DR, the knowledge of the underlying mechanisms, is still insufficient and therapeutics are limited. More studies are needed to provide further insight into the mechanism, which may then lead to new treatments. Especially, identifying early features of the complications is important to interrupt pathologies before neuronal damage starts, to prevent visual impairment. The upregulation of numerous mediators, both angiogenic and inflammatory, has been implicated in the pathogenesis of microvascular retinopathy and will be summarized in this review.

## 2. The Retina

**Figure 1:**
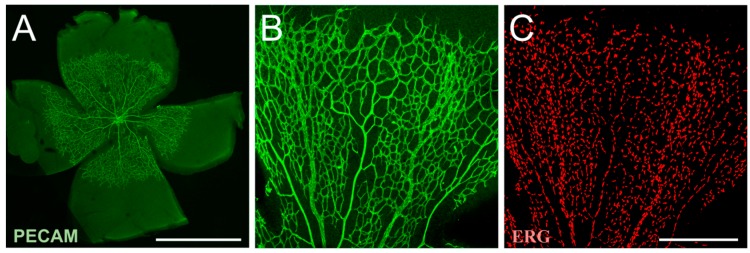
The mouse retina is a model system to analyzing angiogenesis. The mouse retina is a robust tool to analyze in vivo angiogenesis. The avascular retinal vasculature develops gradually as vessels start to grow into the tissue. Starting at postnatal day 0 (P0), blood vessels branch out in a single plane until approximately P8, which allows monitoring of the vessel growth in an intact system including endothelial cells, pericytes, neurons, microglia, astrocytes and Müller cells. Genotype-specific alteration can be analyzed, which may provide insight into molecular and cellular mechanisms of the regulation of angiogenesis. (**A**) Overview of the retina vasculature stained with the endothelial cell marker platelet endothelial cell adhesion molecule (PECAM; CD31) at P5. The retinal leaves show the branching of the multiple retinal vasculatures. The vessels were stained with (**B**) PECAM and **C** ERG to mark endothelial cells and their nuclei, respectively. Scale bar in (**A**) 500 µm, in (**B**,**C**) 250 µm.

The retina is a transparent layer of neural tissue, which can be divided into nine layers, and consists of different cell types including neurons, macroglia, microglia and vascular cells (Figure 1). The function of the retina is the conversion of captured light into electrical energy and the transmission of the resulting action potential to the occipital lobe of the brain. The retina originates from the optic cup, a part of the embryonic diencephalon and, thus, is part of the CNS [27]. 

The construction of the retina might contribute to its vulnerability to diabetes and to the development of DR. There are three structural conditions which are needed to ensure undisturbed light transmission, but subsequently increase the vulnerability of the retina. First, the retina contains a low density of blood vessels, because these would absorb light and interfere with the retinal function. The oxygen tension declines from the inner to the outer retina [28,29,30]. Thus, the retina relies on anaerobic respiration, which is less efficient than aerobic respiration [31]. This is especially problematic because the neurons in the retina have a relatively high metabolic demand. High amounts of ATP are consumed for phototransduction, to maintain ion gradients across the cell membrane, for neurotransmission and to maintain functional photoreceptors and their transient outer segments [32]. Second, axons in the retina are not myelinated since lipids in the myelin sheath would interfere with light transmission; however, unmyelinated nerves require even more energy to maintain their membrane potentials. Third, the number of mitochondria in the retina is relatively low, because they contain light-absorbing heme-based cytochrome proteins. The combination of high metabolic demand of the retinal neurons and low vascular supply, together with few mitochondria, increases the vulnerability of the retina and reduces the ability of the retina to acclimate to metabolic stress situations.

Lactate, a product of anaerobe respiration has been linked to metabolic signaling and retinal neurodegeneration [33,34,35]. Lactate produced in Müller cells and astrocytes is secreted into the subretinal space and can bind to Gpr81 receptors and monocarboxylate transporters, located on the membrane of retinal pigment epithelium, ganglion cells and photoreceptors [33,36,37]. In neurons, lactate is believed to increase the utilization of glucose [38] and it was shown to be neuroprotective in models of cerebral ischemia [38,39] and traumatic brain injury [40,41], whereas lack of lactate has been implicated with neurodegeneration [42]. Another vulnerable structure of the retina is the blood barrier. The retina, as part of the CNS, has a blood–retina barrier (BRB), which is formed by tight junctions between adjacent endothelial cells, protects the neural tissue from various circulating components of the blood and enables the retina to regulate its own extracellular chemical composition [43,44]. The interaction of blood vessels, astrocytes and ganglion cells induces the expression of tight junction proteins, such as occludins, claudins and zonula occludens (ZO) proteins, which are important to maintain the BRB [45,46]. Disruption has been linked to numerous diseases, such as stroke and brain tumors and can lead to edema.

## 3. Diabetic Retinopathy

DR is a severe ocular complication of diabetes mellitus, which is accompanied by hyperglycemia, leukostasis, microvascular damage, inflammation, vascular permeability, occlusion, ischemia and neurodegeneration, all together resulting in blindness.

### 3.1. Hyperglycemia in Diabetic Retinopathy

High blood sugar levels in diabetes mellitus is most probably the causative etiology for DR [47,48]. One of the first events in DR is the glucose-mediated microvascular damage, which further results in increased polyol and hexosamine pathway flux, cellular oxidative stress, activation of protein kinase C, superoxide overproduction by the mitochondrial electron transport chain as well as the increase and activation of advanced glycation end products, advanced lipoxidation end-products and oxidized low density lipoproteins and their receptors [49,50,51,52,53,54,55,56], followed by massive production of free radicals in mitochondria, which further increase oxidative stress and have severe consequences such as chronic inflammation.

Accumulation of these mediators in the retina can trigger further steps, including microglia activation [57,58]. In parallel, hyperglycemia induces microglia activation via reactive oxygen species (ROS) [59]. Generation of ROS induces nuclear factor kappa-light-chain-enhancer of activated B cells (NFκB) nuclear translocation and extracellular signal–regulated kinase (ERK) phosphorylation in microglia [60,61,62,63] and hence production of cytokines, such as tumor necrosis factor (TNF)α, interleukins (IL)-1β, IL-6, IL-8, vascular cell adhesion molecule (VCAM-1) and intracellular adhesion molecule 1 (ICAM-1) [64,65,66].

Hyperglycemia can also influence pericyte loss in early stages of DR, a process which is accompanied by ATP release [67]. In this context, the purinergic P2X7 receptor gained interest, which is involved in regulation of the lumen diameter through ATP signaling [68]. Blocking the purinergic P2X7 receptor in high glucose conditions reduced retinal cell death and the inflammatory response [69].

### 3.2. Leukostasis in Diabetic Retinopathy

Early in diabetes, endothelial cells start to increase the expression of ICAM-1 and P-selectin [70]. Two possible positive activators of ICAM-1 are the microglia-derived cytokines TNFα and IL-1β, suggesting that inflammation might start prior to leukocytosis. ICAM-1 (CD54) is a ligand of the β2 integrin lymphocyte function-associated antigen-1, which is part of a family of leukocyte integrins that are characterized by their β- (CD18) and α-chains (CD11a). ICAM-1 is expressed on the leukocyte surface and enhanced expression of ICAM-1 increases the adherence of leukocytes to the vascular membrane. Binding to CD11/CD18 on the surface of endothelial cells activates leukocytes, such as neutrophils and monocytes and results in accumulation of these immune cells on the luminal vascular surface [71]. This accumulation benefits occlusions, non-perfusion of the retinal vessels and dysfunction of the BRB [72]. Knock-down of adhesion molecules could prevent leukostasis in animal models [73,74]. Leukostasis, among others, is responsible for disruption of the BRB, death of endothelial cells [75] and is an early feature of DR in rodents, emerging within weeks of the onset of hyperglycemia; it has also been described in patients [72,73,75].

Additionally, by binding to β2 integrin lymphocyte function-associated antigen-1, ICAM-1 activates a key adhesion pathway, leading to upregulation of inflammatory cytokines [76,77,78] and promotes an inflammatory cascade in the retina [75]. Animal studies found that retinal leukostasis was decreased in diabetic mice deficient for TNFα. In these mice, a reduction of vascular leakage three and six months after onset of diabetes and a reduction of neurodegeneration after three months of diabetes was detected [79]. However, the deficiency of TNFα did not reduce leukostasis at earlier time points (four and six weeks of diabetes), suggesting that earlier time points were not dependent on TNFα signaling [79]. The temporal relationship between leukostasis and inflammation needs to be further investigated to determine the exact mechanisms.

Beside the increase in inflammation, leukostasis also leads to non-perfusion areas in the retina causing tissue death and neurodegeneration. However, dying neurons could contribute to vascular closure and thus, also in this relation, the temporal relationship still needs to be studied.

## 4. Microglia

Microglia, the macrophages of the CNS, are derived from hematopoietic stem cells [80,81]. Amoeboid, mesodermal myeloid progenitors enter the retina during development and differentiate into ramified parenchymal microglia in the adult retina [80,82,83,84]. These glia represent 5–12% of the cells in the CNS and can be identified by their immunoreactivity for CD45 (pan-leukocyte), MHC class I, and MHC class II [85,86]. Microglia are a population of self-renewing, relatively long-lived, innate immune cells [87,88].

For a long time, resident ramified microglia were described as quiescent cells, waiting for their activation, but today it is known that ramified microglia are involved in multiple processes and contribute to tissue and neuronal homeostasis [89,90,91]. Microglia have protective and corrective properties, for example, microglia are in close contact with neurons, transiently contacting synapses with their processes to monitor their functional state [92] and synapse stability, ensuring healthy vision [93]. The processes of microglia are constantly in motion, extending, retracting and continuously scanning their microenvironment [94]. Additionally, neurons express microglia ligand proteins such as fractalkine on their surface, which can be specifically recognized by microglia surface receptors, such as fractalkine receptor. Thus, on one hand these interactions contribute to controlling microglia function and on the other hand, microglia themselves can sense neuronal changes and promptly react to very subtle changes in their microenvironment [94,95].

The activation of microglia is determined by extracellular signals, including neuronal damage, chronic neurodegeneration, dying cells, extracellular liposaccharides and nucleic acids, which are recognized by a broad range of receptors [82,96], such as toll-like receptors (TLR) and receptors of advanced glycation end products. These enable microglia to detect pathogens via signaling of the pro-inflammatory nuclear transcription factor NFκB [97]. Translocation of NFκB is followed by production of cytokines and other inflammatory mediators.

Upon activation, ramified microglia undergo a series of stereotypic, morphological, phenotypic, and functional changes [98]. During activation, microglia start to proliferate and change their morphology from ramified state, with long and thin processes, to amoeboid state with larger cell bodies and thicker and shorter processes [86,95,99,100,101]. Additionally, immunoreactivity and migratory properties of microglia are enhanced [95,100,101]. Thus, expression levels of pro- and anti-inflammatory mediators (interleukins, cytokines, chemokines, proteases, nitric oxide and ROS) are increased as is the phagocytic activity of microglia [102,103].

In vitro studies showed that microglia and macrophages can become both pro- (M1) and anti-inflammatory, but pro-healing (M2) [104,105]. M1 microglia are induced by Th-1 cytokines, interferon γ or lipopolysaccharide and express high levels of IL-12, IL-23, TNFα, IL-1β and IL-6 [106]. These cells are neurotoxic. M2 microglia are induced by Th-2 cytokines, such as IL-4, IL-10 and IL-13 and express high levels of IL-10 [106]. These cells are mainly phagocytic and their response is neuroprotective. Phagocytic microglia are responsible for clearance of cell debris and necrotic or apoptotic cells. However, in vivo, microglia had an intermediates phenotype [104,107], suggesting that the distinction between M1 and M2 microglia is hazy and that microglia can adapt their phenotype to meet demands [3,108].

Microglia activation is a highly regulated process; the amount is determined by the affected tissue and the extent of dysfunction, damage or infection [109,110]. Normally, activation of microglia has protective properties, but under certain conditions could also result in damage to the CNS. Dysregulation of microglia activation may result in severe complications, including vascular breakdown, glia dysfunction and neuronal death [111]. Furthermore, dysregulation was associated with many diseases as well as neurodegeneration [112,113], suggesting that activation could become maladaptive in certain conditions.

Involvement of microglia was described in most CNS disorders and progression and healing of these diseases highly depends on microglia activation [114]. Changes in glia already occurred in initial stages of the disease.

### 4.1. Microglia in the Retina

As part of the CNS, retinal microglia adopt the role of tissue macrophages. In the adult retina, ramified microglia are mainly located in the inner retinal layers, such as the nerve fiber, the ganglion cell and the inner and outer plexiform layers [87,115,116], whereas they were barely found in the inner nuclear and totally absent in the outer nuclear layer [86,99].

In the healthy retina, microglia are required for normal retinal growth, the immune system, neurogenesis, synaptic pruning, controlling development, formation of blood vessels, aging and retinal function via interaction with neurons, glia and endothelial cells and secretion of growth factors and cytokines as well as neuroprotective and anti-inflammatory mediators [117,118,119,120,121,122,123]. In pathogenesis of the retina, microglia play a role in infection, trauma and retinal detachment.

### 4.2. Inflammation in Diabetic Retinopathy

The correlation between inflammation and DR was discovered when diabetic patients treated with salicylates for rheumatoid arthritis had fewer complications than untreated patients [124]. Since this discovery, increasing evidence showed that inflammation indeed plays an important role in DR; now, it is known that the diabetic retina always present a low chronic level of inflammation [125,126]. In diabetic patients, this low-level inflammation may be tolerated for years without any damage, however, mounting diabetic alternations may increase inflammation over time and it becomes more severe and chronic.

Inflammation is already associated with many other retinal diseases [127] and in DR, inflammation might be induced by leukocyte adhesion to the retinal vasculature and alteration of the BRB. A first step might be perivascular accumulation of activated microglial cells.

### 4.3. Microglia Activation in Diabetic Retinopathy

For a long time, microglia were underappreciated in most studies of DR. However, in recent years, numerous studies showed that microglia indeed play a significant role in DR. Microglia are altered by hyperglycemia, ischemia, hypoxia, dyslipidemia and endoplasmic reticulum stress, but the exact mode of microglia activation in DR is still unknown [125,126,128].

**Figure 2:**
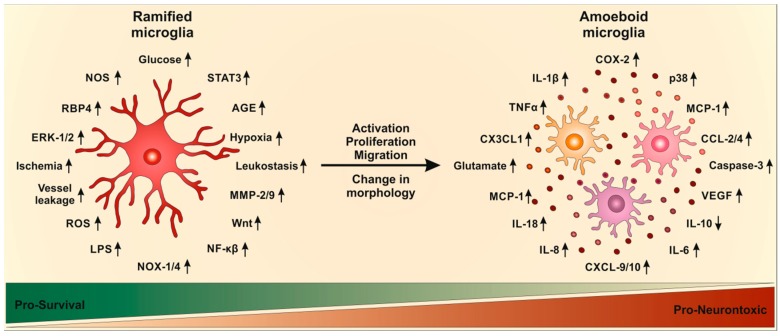
Possible mechanisms underlying microglial activation and subsequent inflammatory responses in diabetic retinopathy. Ramified microglia are influenced by hyperglycemia, alterations in ischemia, vessel leakage and upregulation of various mediators. Microglia activation means morphological changes, proliferation and migration. This activation results in an inflammatory response, including downregulation (down arrow) of cytokine IL-10 and growth factors as well as upregulation (up arrow) of various cytokines, chemokines and neurotoxins.

The activation of microglia in DR has also been documented in patients. DR is accompanied by an increase in cytokines, which further increase activation of microglia resulting in uncontrolled microglia activation, which is involved in neurotoxicity and tissue damage. Hyperglycemia induced TLR-2, TLR-4 and NFκB expression via an increase in ROS [129], indicating that oxidative stress plays a major role in microglia activation. Decreasing oxidation stress, e.g., via VP10/39 would be a promising therapeutic for DR [130]. The activation of NFκB leads to further production of cytokines and other inflammatory mediators [97]. NFκB was increased in activated microglia after hypoxia induction and was required for retinal angiogenesis [65]. Pathways of microglia activation in DR are summarized in Figure 2.

Hypoxia-inducible factor-1 (HIF-1) is the key player for the cellular response to reduced oxygen levels [131,132]. The HIF-1α subunit is stabilized in hypoxic conditions, binds to the HIF-1β subunit and thereby activates transcription of target genes, which among others are involved in proliferation, angiogenesis and cell survival [133,134,135,136]. Especially in highly active photoreceptors HIF-1 is of highest importance. It was reported to be constitutively active [137] and protects against retinal damage [138]. HO-1 is a target product of HIF-1α and activation of the Nrf2/HO-1 pathway to reduce oxidative stress could be an interesting approach to treat DR [139].

ERK phosphorylation is also involved in microglia activation [140] ERK can be activated by different signals and may have different consequences, e.g., ROS induced phosphorylation of ERK is important for TNFα expression [63] and vascular endothelial growth factor (VEGF) mediated activation of ERK is important for endothelial cells survival and proliferation [141,142]. LPS treatment in human retinal pigment epithelial cells activated ERK signaling, which is required for cytokine gene transcription [143]. Furthermore, ERK activation is important to induce expression of IL-6, MCP-1, and ICAM-1 [62]. High glucose levels in the retina were reported to increase phosphorylation of ERK [144] and also streptozotocine-induced diabetic rats showed an increase in proliferation and ERK expression in the retinal pigment epithelium [145].

Activation of microglia in the retina involves proliferation, migration and changes in their morphology. In streptozotocin-induced diabetic rats, the morphology of microglia changed from ramified to amoeboid [26,146]. The number of microglia increased in DR, suggesting that proliferation or immigration is enhanced [118]. Microglia, which are normally absent in the outer nuclear layer, migrated into the outer plexiform layer and the photoreceptor layer, while the amount of microglia in the ganglion cell layer decreased [26,86,99,147]. In another study, the density of microglia was not enhanced in diabetic rats, but the amount of activated microglia was increased [148]. Whether microglia activation in DR is neuroprotective or neurotoxic, is currently under discussion [149,150,151,152,153]. In the rodent retina, microglia activation started 1 month after diabetes induction and microglia invaded the inner plexiform layer after four months. After 14–16 months, microglia were found to migrate into the outer nuclear and photoreceptor layers [14,26,147].

In the human retina, microglial activation was present at distinct stages of DR. The number of microglia was increased and these cells migrated into the inner retinal layers and cluster around micro-aneurysms and intra-retinal hemorrhages. In DME, high numbers of microglia were found throughout the retina and in the sub-retinal space [118]. In retina of patients with NPDR, microglia migrated into the plexiform layers and increased in number, whereas in PDR, microglia significantly increased in number and clustered around ischemic areas [118,154].

## 5. Molecular Pathways of Inflammation in Diabetic Retinopathy

A complex chain of mechanisms, mediators and signaling cascades contribute to inflammation in DR. These include inflammatory cells, such as microglia and neutrophils, as well as inflammatory mediators, such as cytokines, chemokines, neurotoxins, growth factors and adhesion molecules [14,28,155,156,157,158,159]. The majority of the currently described mediators are summarized in Table 1.

The inflammatory process starts early in DR and can become chronic [28,158,159,160,161]. Chronic inflammation, characterized by prolonged duration and tissue destruction, induces a pro-inflammatory shift and enhances neuro-inflammation [162]. These characteristics apply to development and progression of DR [163,164,165]. Chronic inflammation has already been implicated with other, age-related retinal diseases such as age-related macular degeneration and age-induced changes in microglia [166].

Initially, upregulation of growth factors and cytokines may provide neurotrophic signals to maintain neuronal function and support retinal cell survival. However, over longer periods enhanced expression of chemokines and cytokines are maladaptive and cause vascular damage, DME and neovascularization [167].

### 5.1. Cytokines

Increased levels of cytokines, including IL-1β [28,168,169,170,171], IL-6 [172,173], IL-8 [173], VEGF [177] and TNFα [28,168,178,179] have been found in diabetic patients and rodent models of diabetes [198,199,200]. The levels of TNFα and IL-8 are further increased with progression of the disease; the highest amounts were measured in PDR [201,202,203]. In rodent diabetes models, the increase in IL-6 and TNFα is maintained up to 24 weeks of age [204]. Induction of diabetes in rats using streptozotocin increased retinal IL-6 and TNFα, whereas anti-inflammatory IL-10 was decreased, suggesting a pro-inflammatory and neurotoxic shift [146]. There was a local increase in IL-6 in the vitreous humor of diabetic patients, which was correlated with progression of DR [205].

### 5.2. VEGF

VEGF was identified based on its vascular effects, but was later also described as an important cytokine and signaling molecule for neurons. VEGF is neuroprotective in peripheral and central neurons [206,207] and is induced by hypoxia through the transcription factor HIF-1 [208]. It caused changes in tight junction organization and increases vascular permeability, via protein kinase C [44,209,210]. Deficiency of the VEGF-A gene in Müller cells reduced the effects of diabetes in the retina and decreased leukostasis, inflammation and vascular leakage, suggesting that Müller cells play an important role in retinal inflammation [211].

### 5.3. TNFα

Enhanced levels of TNFα and cyclo-oxygenase-2 are detected early in DR [180] and TNFα expression interferes with progression of DR. TNFα and IL-1β positively influenced ICAM-1, suggesting that TNFα could be involved in leukostasis. CD40, a member of the TNF receptor family, is normally upregulated in the rodent diabetic retina. Blocking CD40 reduced ICAM-1 upregulation, retinal leukostasis and degeneration capillary [212]. TNFα also exerts a crucial role in BRB breakdown [213] and neuronal death, by increasing caspase-3 expression [214]. Blocking TNFα receptor TNFR1, not only decreased vascular alternation, but also reduced neuronal cell death [214].

### 5.4. Chemokines

Enhanced levels of chemokines, such as CCL-2, CCL-4, CXCL-9 and CXCL-10, were measured in vitreous samples of patients with PDR [181]. Additionally, DR increases the levels of chemokine monocyte chemoattractant protein (MCP)-1 [28,173,182], which is involved in recruiting and activating microglia and leukocytes as well as in fibrosis and angiogenesis [215]. In streptozotocin-induced diabetic rodents, upregulation of MCP-1 started during the initial stages of DR and increased further with disease progression [216]. The main sources of MCP-1 are neurons and MCP-1 is involved in microglia activation by inducing TNFα release from microglia via the p38 and ERK pathway [63]. ERK1/2 and p38 phosphorylation was increased in microglia of diabetic rats, which was associated with harmful microglia activation [217,218].

### 5.5. Novel Molecular Targets in Diabetic Retinopathy

Over the last years, intensive research identified several new mediators influencing inflammation and neurodegeneration in DR. These new signaling pathways will be important to identify novel and better therapeutics.

Retinol-binding protein 4 (RBP4) expression was enhanced in patients with diabetes and its increase was correlated with retinal neuronal degeneration, early-onset of microglia activation and increased expression of pro-IL-18 and activated IL-18 [174,175,176]. Neurodegeneration in RBP4 overexpressing mice was shown to be independent of retinal microvascular pathology, suggesting an independent pathway in which microglia could influence neurodegeneration. RBP4 might alter inflammation via TLR4, c-Jun N-terminal kinases and p38 mitogen-activated protein kinase signaling pathways [176].

Another novel mediator of inflammation in DR could be nicotinamide adenine dinucleotide phosphate (NADPH) oxidases (NOX)-1/4. Inhibition of NOX1/4 with GKT137831 not only reduced leukocyte adherence to the vasculature and vascular leakage, but also hypoxia-induced ROS levels and the pro-inflammatory phenotype (M1) of microglia and macroglia [188], suggesting that NOX inhibitors might be a promising therapeutic target in DR.

CX3CL1 was involved in activation of microglia in mouse models of type 1 diabetes [183]. CX3CL1 is a neuronal, membrane-bound chemokine, which could be proteolytically cleaved, became soluble and activated the CX3CR1 receptor in microglia. CX3CL1 deficiency in diabetic mice increased levels of IL-1β, negatively influenced the number of neurons in the ganglion cell layer and positively influenced the number of microglia cells [183]. In a model of retinitis pigmentosa, a progressive degenerating disease, deletion of CX3CR1 receptor caused an increase in microglial infiltration into the photoreceptor layer and accelerated photoreceptor apoptosis via increasing phagocytosis [219]. The role of CX3CR1 in DR was also analyzed in mice with streptozotocin-induced diabetes [220]. Here, CX3CR1 deletion expedited the onset of DR and increased apoptosis in the retina. In mouse models of mouse type 1 diabetes, loss of CX3CR1 signaling led to increased systemic inflammation and perivascular clustering of proliferating microglia with increased IL-1β expression [221]. Thus, CX3CR1 signaling bears a protective effect in the diabetic retina and might provide effective and successful means for treating DR.

A study on bone marrow (BM)-derived pro-inflammatory monocytes and BM-derived reparative circulating angiogenic cells showed that altering acid sphingomyelinase-mediated sphingolipid signaling in BM-derived cells could normalize inflammation in DR [222]. Acid sphingomyelinase was significantly increased in retinal endothelial cells from diabetic patients. [223], suggesting that by decreasing sphingolipid signaling, inflammation in DR could be reduced.

Matrix metalloproteases (MMP) were also analyzed in the context of DR, because their levels were increased. Especially MMP-2 and MMP-9 levels were enhanced in patients and in animal models of diabetes [189,190,191,192]. MMPs play an acute role in inflammation and activation of chemokines. Elevated levels of glucose could induce MMP-2 expression [224]. Treating streptozotocin-diabetic rats with minocycline, an inhibitor of MMP-1 and MMP-9, together with a cyclooxygenase (COX) and tPA inhibitor prevented development of DR [193]. Furthermore, MMP-2 was sensitive to oxidative stress, upon increased superoxide levels, MMP-2 was activated and increased apoptosis of retinal capillary cells in diabetic rats [194]. Thus, inhibition of MMP-2 could be another promising therapeutic target in DR.

STAT3 signaling was previously connected to cytokine signaling in vascular inflammation. IL-6 induced STAT3 signaling and thereby increased retinal endothelial permeability and vascular leakage. This was accompanied by VEGF-induced reduction in ZO-1 and occluding, two tight junction proteins [195]. miR-146a reduced IL-6/STAT3/VEGF signaling in high glucose conditions and overexpression of this microRNA decreased apoptosis, suggesting that miR-146a is a potential target for reducing inflammation and degeneration in DR [196].

Furthermore, aberrant activation of Wnt signaling played a pathogenic role in DR [197]. Levels of β-catenin were increased in diabetic patients and animal models of diabetes [197]. In vitro, Wnt activation using β-catenin treatment in ARPE19 cells, a cell line derived from human retinal pigment epithelium, increased the expression of VEGF, NFκB and TNFα, as well as the generation of ROS [225]. Thus, blocking Wnt signaling could be a potential therapeutic target in treating DR by reducing inflammation. Indeed, several studies reported a reduction in pathology of DR after inhibition of Wnt signaling [226,227,228]. Inhibition of Wnt using DKK-1 reduced retinal inflammation in diabetic rats [229], while in patients with DR, serum levels of DKK-1 were reduced [230], indicating that there could be increased Wnt signaling in DR patients.

## 6. Neurodegeneration in Diabetic Retinopathy

The retina comprises four types of neurons: photoreceptors (rod and cones), bipolar cells, amacrine cells and ganglion cells, which are involved in photo transduction, modulation and signal transfer. The encoded visual signal is transduced to the brain through axons of the ganglion cells. Impairment of neurons in the retina may result in impairment of vision.

In DR, neuronal defects are among the earliest detectable changes, resulting in a retinal neuropathy [19]. Neuronal cell death occurs in cultured retinas [17], diabetic mice [231] and patients [232]. DR is always associated with impaired neuronal function. Thus, treatment must interfere not only with vascular alterations and inflammation, but also with neuronal defects. Loss of neurons in the retina starts even before development of clinical symptoms [233,234] and neuro-retinal function is weakened before vascular lesions [235,236,237,238,239].

Retinal ganglion neurons are the first to die in DR and thus, loss of their nerve fibers in the nerve fiber layer occurs [18,233,240,241,242,243]. A reduction in ganglion cells was described in both diabetic mice [19] and patients [18,244]. In animal models of diabetes retinal ganglion, cell loss started as early as 5 weeks after induction of hyperglycemia [245]. *db*/*db* diabetic mice developed neurodegeneration in the retina, starting at the age of eight weeks [104,204,246].

Neuronal cell death in DR is due to an increase in apoptosis [247,248]. The loss of neurons results in thinning of the inner retinal layers and the nerve fiber layer in diabetic mice [18,233,249,250,251]. In these mice, ganglion cell loss occurred but there was no difference in the density of pericyte or acellular capillaries, suggesting that neurodegeneration preceded the established clinical and morphometric vascular changes. Additionally, the remaining ganglion cell bodies began to swell and increased in size [243]. In patients with diabetes and no to minimal DR, there is significant, progressive loss of the nerve fiber layer, the ganglion cell layer and the inner plexiform layer [249].

Electroretinography (ERG) can be used to measure neuronal defects in DR patients. The oscillatory potential implicit time gives information about the electrophysiological communication between neuronal cells. ERG measurements detect local abnormalities or widespread pathology, even in very initial stages of the disease. In DR, patients show a reduction in electric activity [252].

## 7. The Influence of Microglia on Neurodegeneration in Diabetic Retinopathy

Activated microglia are strongly involved in neurodegeneration [28]. Production of neurotoxic factors, such as glutamate, oxidative stress, caspase-3, MMPs and nitrous oxide, which are all neurotoxic mediators, result in neuronal cell dysfunction as well as damage to pericytes and endothelial cells. An imbalance in retinal production of neuroprotective mediators and pro-inflammation cytokines was involved in the development of neurodegeneration in DR [185].

Glutamate is toxic to retinal ganglion neurons [204] and extracellular glutamate led to over-activation of ionotropic glutamate receptors, mainly α-amino-3-hydroxyl-5-methyl-4-isoxazole-propionate (AMPA) and *N*-methyl-d-aspartame (NMDA) receptors, which resulted in uncontrolled intracellular calcium responses and cell death [253,254]. Oxidative stress originates from aberrant production of mitochondria-derived ROS and super oxide induced by hyperglycemia [255]. Hyperglycemia-induced downregulation of neurotrophic mediators such as nerve growth factor (NGF), pigment epithelium-derived factor (PEDF), iron-responsive element-binding proteins (IRBP) and somatostatin, also contributed to neurodegeneration [184].

## 8. Microvascular Pathology and Defective BRB Integrity

Microvascular pathologies have been implicated in DR since the discovery of the disease. For a long time, many DR studies in both clinic and animal models focused on vascular dysfunction, including impaired endothelial cells, death of pericytes, thickening of retina capillary basement membrane and altered tight junctions [256,257]. Vascular changes are caused by leukostasis [258], microthrombosis [259] or invasion of Müller cells into the vascular lumen [260].

In the healthy retina, vascular endothelial cells and pericytes are responsible for nutrient supply, waste product removal and constitute the BRB. In DR, increased capillary permeability and capillary occlusion are the major pathologies to identify the complication in diabetic patients and categorize the state of disease progression.

DR progression is defined by a decrease in retinal perfusion and disruption of the BRB [261]. Changes in retinal blood vessel permeability reduced the quantity of occludin in retina endothelial cells, which caused disorganization of tight junction proteins and thickening of the vascular basal membrane [75,262,263]. Phospholipases A2 was shown to increase early in DR and was linked to BRB alterations [264]. Apoptosis was increased in pericytes and endothelial cells [265,266]. The loss of pericytes, caused by insulin response modifications, had severe consequences for the retinal vasculature such as unstable retinal perfusion, capillary hyper-perfusion, hematoretinian barrier abrogation, appearance of capillary dilation, micro-aneurysm formation, occlusion of capillaries, retinal ischemia, increased in vascular permeability and endothelial cell degeneration [267,268].

Leakage of the BRB allows serum proteins, such as circulating cytokines and chemokines, as well as high glucose levels and advanced glycation-end products into the retina parenchyma and thus additionally contribute to activation of microglia and immune cell infiltration into the retina. Thus, through disruption of the BRB, the retina is affected by both external and internal signals [63,269,270]. BRB disruption could also increase the production of neurotoxic glutamate and contribute to neurodegeneration [271].

## 9. Angiogenesis and Inflammation

Angiogenesis is the course of endothelial cell migration, proliferation, vessel formation and remodeling of the vascular system. The formation of new vessels from existing ones depends on degradation of the extracellular matrix. Angiogenesis is an extremely regulated process which vascular alteration in DR can disrupt resulting in uncontrolled sprouting.

In DR, neovascularization is caused by an imbalance of pro-angiogenic mediators and ischemia resulting in abnormal growth of new vessels, which interferes with the normal function of the retina, namely light transmission. The consequences are leaky vessels and an accumulation of fluids and proteins.

Angiogenesis and inflammation are not independent, but rather two interacting processes which share several mediators (e.g., VEGF) and signaling pathways. Thus, microglia might induce neovascularization by releasing pro-angiogenic mediators, including cytokines, growth factors and proteases [272,273]. Cytokines could directly or indirectly enhance vascular growth in endothelial cells [274,275,276]. Moreover, endothelial cells could produce pro-inflammatory molecules [277,278].

The major regulator and pro-angiogenic factor is VEGF, which was increased after hyperglycemia and hypoxia [279,280]. An imbalance in the expression of VEGF is responsible for the increased neovascularization in DR. Thus, altering VEGF expression is a major therapeutic target in DR. Angiopoietin-2 is also an important modulator of angiogenesis and a regulator of the BRB [187] which exhibited increased levels in patients with DME [186]. However, both mediators are also involved in inflammation and might act as pro-inflammatory mediators, further increasing microglia activation and expression of inflammatory cytokines [281,282].

## 10. Interaction of Microglia with Macroglia in the Retina

The retina contains two types of macroglia: Müller cells and astrocytes. Müller cells are important for the regulation of retinal metabolism and modulate neuronal and blood vessel function [46], whereas astrocytes provide nutritional and regulatory support.

In DR, Müller cells increased the expression of glial fibrillary acidic protein, experienced altered ability to convert glutamate into glutamine because they express less glutamine syntheses and their number also increased [147,283,284], suggesting, that Müller cells are strongly involved in neurodegeneration via glutamate signaling. Microglia could directly influence Müller cells, which respond to microglia activation on the molecular and functional levels. Via bidirectional signaling between the two cell types, the activation and migration of microglia was further increased. Müller cells increased the inflammatory response across the retinal layers by chemotaxis and adhesive cell contacts and thus increased the mobilization of migratory microglia [285].

In the healthy retina, astrocytes are only located in the nerve fiber layer and surround blood vessels as well as ganglion cells. In DR, astrocytes became activated, change their morphology, proliferate, migrate and secreted pro-inflammatory mediators such as IL-6, MCP-1 and VEGF [144,286]. The translocator protein, a biomarker for microglial and astrocyte gliosis in brain degeneration, was upregulated in retinal microglia during retinal inflammation and injury. In astrocytes, the endogenous ligand of translocator protein, diazepam-binding inhibitor was upregulated, suggesting an astrocyte-microglia interaction [287].

## 11. Treatment of Diabetic Retinopathy by Altering Microglia

Treatment of DR should be targeted at the first event of the complication. However, investigations about the exact temporal coherencies are still in progress. Cytokine upregulation, leukostasis and glia activation were among the first symptoms of DR and would be ideal targets for therapy to prevent downstream neurodegeneration.

### 11.1. Photocoagulation

Developed in the 1950s, photocoagulation was one of the first treatments for DR and is still standard of care. Via prolonged exposure to bright light, a reduction of neovascularization and DME was precipitated, together with microglia activation [288,289,290]. The microglia response was prolonged after light exposure and expression of antigens, such as CD11b, CD45, and F4/80, was induced.

Following photocoagulation, morphological changes from ramified to amoeboid microglia occurred and these microglia migrated into the outer nuclear layer and to the laser-induced injury [272,291,292]. Activation and infiltration into the outer nuclear layer started 6 h after light induction, but the number of amoeboid microglia declined 7 days after light exposure and the cells were then present mostly in the sub-retinal space [293]. Microglia activation peaked parallel to the apoptotic loss of cells 1 day after injury [293]. Expression of chemokines, pro-angiogenic mediators and inflammatory cytokines increased after laser-induced injury [272,293].

Song et al. (2017) showed that deletion of anaphylatoxin C5a receptor (C5aR), a receptor previously associated with age-related macular degeneration, reduced the light-induced migration of microglia. After exposure to light, C5aR mRNA was increased as was the number of Iba-1 positive microglia cells. Microglia cells expressed receptors for C5a, which promoted recruitment of microglia and macrophages [294].

### 11.2. VEGF Blocking

Blocking VEGF had inhibitory effects on vascularization, but was also suspected to have neurotoxic effects [295], however these effects are still under discussion. Beneficial effects of the treatment have been documented [296,297] and it seems that VEGF has a dual role in neuroprotection and neovascularization in hypoxic regions. Diverse isoforms of VEGF are currently used in the clinic. Furthermore, the PKCβ/HuR/VEGF pathway has been suggested as a potential pharmacological target for DR.

Inhibition of the receptors for VEGF, VEGF receptor 1 and/or 2, blocks retinal microglia migration and infiltration after laser-induced choroidal neovascularization [298]. Choroidal neovascularization is a serious complication of age-related macular degeneration. Zhou et al. (2017) showed that the number of M1 and M2 macrophages increased following neovascularization. M1 macrophages (CD80-positive cells) were most prominent in the choroid-retinal pigment epithelial complex, whereas the M2 macrophages (CD206-positive cells) were mostly increased in the retina itself [299]. Blocking VEGF with a neutralizing antibody significantly inhibited diabetes-related vascular leakage, leukostasis, expression of ICAM-1, abnormal localization and degeneration of the tight junction protein ZO-1, as well as the cell adhesion protein vascular endothelial cadherin [300]. Furthermore, expression of several cytokines and chemokines were decreased after blocking VEGF [300], suggesting that blocking VEGF might be a possible target for the reduction of microglia activation.

### 11.3. Steroid Therapy

Glucocorticoids and their pharmacological derivates are anti-inflammatory and anti-angiogenic mediators, which are already used for treatment of DME and PDR, but are only a second-choice treatment for patients with suboptimal response to anti-VEGF treatment [301,302]. Glucocorticoids such as the synthetic triamcinolone reduced the expression of VEGF, promoted the stability of the BRB, prevented photoreceptor degeneration, inhibited activation of microglia and Müller cells and reduced expression of TNFα and the activation of p38/SAPK signaling pathways [303,304,305]. Glucocorticoids had anti-inflammatory properties, including the inhibition of NFκB and induction of the phagocytic activity of monocytes and macrophages [306,307,308]. Glucocorticoid treatment might be beneficial for treatment of DR, by affecting both vasculature and neurons. However, the exact role of steroids in the treatment for DR and DME remains to be fully elucidated.

### 11.4. Direct Prevention of Microglia Activation

Blocking glial cell activation might be a strategy to prevent neurodegeneration in DR and inhibition of microglia activation has been recently investigated in clinical trials. There are two tetracycline antibiotics in the focus of research: minocycline and doxycycline.

Minocycline is a semisynthetic, second-generation tetracycline antibiotic with anti-inflammatory properties, which prevents microglia activation and is neuroprotective in mice [28,309,310]. Treatment with minocycline resulted in reduced production and release of inflammatory cytokines, such as TNFα and IL-1, reduction in the retinal vascular permeability, reduced disruption of tight junctions and reduced activation of caspase-3 [28,311,312]. However, retinal neuronal death was not reduced after minocycline treatment, which raised the question if minocycline alone could be a treatment in DR [311]. In patients with DME, minocycline improved visual function, central macular edema and vascular leakage, and reduced neuropathic pain in diabetic patients [313,314].

Doxycycline, also a semisynthetic, second-generation tetracycline was neuroprotective in mice by reducing cleaved caspase-3 levels and microglial activation [309,315]. In patients with severe NPDR or non-high-risk PDR, doxycycline treatment directly improved foveal sensitivity starting six months after treatment [316]. However, in patients with mild to moderate NPDR, there was no improvement in visual function, suggesting that doxycycline might have different effects at distinct stages of DR.

## 12. Conclusions and Perspectives

In the last years, it became evident that microglia play a key role in DR and chronic inflammation has now been linked to neurodegeneration in the retina. Dysregulation of microglia activation most probably is the major alteration, resulting in a shift from pro-survival to pro-neurotoxic. The temporal components need to be analyzed more closely to identify which alteration is the trigger, and which one is the cause. Thus, whether microglia activation is the cause or the result of neuronal alternations needs to be further investigated; however, the early increase in cytokines points to microglia activation as the catalyst for subsequent neurodegeneration. Furthermore, the mechanism behind microglia activation needs to be explored in-depth. It is still not known if leukostasis or hyperglycemia directly influences the activation of microglia. Focusing more on the role of the disruption of the BRB in microglia activation could also be important, because the leakage is followed by massive invasion of glia cells and further cytokine release. Numerous new molecular mediators were recently identified, suggesting new opportunities for potential therapeutics. Currently, the treatment of DR is insufficient and further studies are needed to evaluate the therapeutic potential of inhibiting microglia activation. Furthermore, treatment approaches involving multiple mechanisms, instead of only one target should be the center of research. When searching for a potential therapeutic, one should always keep in mind the multiple pathways involved and that the retina is a complex structure and interaction of all retinal cells needs to be considered.

## Figures and Tables

**Table 1:** Mediators in diabetic retinopathy, including cytokines, chemokines, growth factors, adhesion molecules, neurotoxins and others. These mediators are either upregulated (up arrow) or downregulated (down arrow) in diabetic retinopathy.

Mediators	Regulation	Relevance	References
Cytokines			
IL-1β	↑	Immuno-stimulation Increased ICAM-1	[28,168,169,170,171]
IL-6	↑	Immuno-stimulation	[172,173]
IL-8	↑	Immuno-stimulation	[173]
IL-18	↑	Immuno-stimulation	[174,175,176]
VEGF	↑	Immuno-stimulation Angio-stimulation Neuroprotective	[177]
TNFα	↑	Immuno-stimulation Increased ICAM-1 Increased leukostasis	[28,168,178,179,180]
COX-2	↑	Immuno-stimulation	[180]
Chemokines			
CCL-2	↑	Immuno-stimulation	[181]
CCL-4	↑	Immuno-stimulation	[181]
CXCL-9	↑	Immuno-stimulation	[181]
CXCL-10	↑	Immuno-stimulation	[181]
MCP-1	↑	Immuno-stimulation Increased fibrosis Angio-stimulation	[28,173,182]
CX3CL1	↑	Immuno-stimulation Neuroprotective	[183]
Growth factors			
NGF	↓	Cellular toxicity	[184]
PEDF	↓	Cellular toxicity	[184]
IRBP	↓	Cellular toxicity	[184]
Somatostatin	↓	Cellular toxicity	[184]
Adhesion molecules			
ICAM-1	↑	Increased leukostasis	[64,65,66]
VCAM-1	↑	Increased leukostasis	[64,65,66]
Neurotoxins			
ROS	↑	Cellular toxicity	[185]
NO	↑	Cellular toxicity	[185]
Glutamate	↑	Cellular toxicity	[185]
Caspase-3	↑	Cellular toxicity	[185]
Others			
LPS	↑	Immuno-stimulation Cellular toxicity	[106]
AGE	↑	Immuno-stimulation Cellular toxicity	[49,50,51,52,53,54,55,56]
LEP	↑	Immuno-stimulation Cellular toxicity	[49,50,51,52,53,54,55,56]
Angiopoietin-2	↑	Angio-stimulation	[186,187]
RBP4	↑	Immuno-stimulation Neurotoxic Increased vascular leakage	[174,175,176]
NOX-1/4	↑	Immuno-stimulation Increased leukostasis Increased ROS Increased vascular leakage	[188]
MMP-2/9	↑	Immuno-stimulation Increased chemokines Neurotoxic	[189,190,191,192,193,194]
STAT3	↑	Immuno-stimulation Increased vascular leakage	[195,196]
Wnt	↑	Immuno-stimulation Increased ROS	[197]

IL: interleukin; VEGF: vascular endothelial growth factor; TNF: tumor necrosis factor; COX: cyclooxygenase; CCL: chemokine (C-C motif) ligand; CXCL: chemokine (C-X-C motif) ligand; MCP: monocyte chemoattractant protein-1; CX3CL: chemokine (C-X3-C motif) ligand; NGF: nerve growth factor; PEDF: pigment epithelium-derived factor; IRBP: iron-responsive element-binding protein; ICAM: intercellular adhesion molecule; VCAM: vascular cell adhesion protein; ROS: reactive oxygen species; NO: nitric oxide; LPS: lipopolysaccharide; AGE: advanced glycation end products; LEP: leptin; RBP: retinol binding protein; NOX: *n*icotinamide adenine dinucleotide phosphate (NADPH) oxidase; MMP: matrix metalloproteinase; STAT: signal transducer and activator of transcription; Wnt: wingless.

## Abbreviations

AMPA	α-amino-3-hydroxyl-5-methyl-4-isoxazole-propionate
C5aR	anaphylatoxin C5a receptor
BRB	blood-retina barrier
CCL	CC chemokine ligands
CNS	central nervous system
CX3CL	chemokine (C-X3-C motif) ligand
COX	cyclooxygenase
DME	diabetic macular edema
DR	diabetic retinopathy
ERG	electroretinogram
ERK	extracellular signal–regulated kinase
HIF	hypoxia-inducible factor
IL	interleukins
ICAM	intracellular adhesion molecule
IRBP	iron-responsive element-binding proteins
MMP	matrix metalloproteases
MCP	membrane cofactor protein
NMDA	*N*-methyl-d-aspartame
NOX	nicotinamide adenine dinucleotide phosphate (NADPH) oxidases
NGF	nerve growth factor
NO	nitric oxide
AGE	advanced glycation end products
LEP	leptin
NPDR	non-proliferating diabetic retinopathy
NF	nuclear factor
PEDF	pigment epithelium-derived factor
PDR	proliferating diabetic retinopathy
ROS	reactive oxygen species
RBP	retinol-binding protein
STAT	signal transducer and activator of transcription
TNF	tumor necrosis factor
VCAM	vascular cell adhesion molecule
VEGF	vascular endothelial growth factor
ZO	zonula occludens
